# Visualizing the strongly reshaped skyrmion Hall effect in multilayer wire devices

**DOI:** 10.1038/s41467-021-24114-8

**Published:** 2021-07-12

**Authors:** Anthony K. C. Tan, Pin Ho, James Lourembam, Lisen Huang, Hang Khume Tan, Cynthia J. O. Reichhardt, Charles Reichhardt, Anjan Soumyanarayanan

**Affiliations:** 1 Data Storage Institute, Agency for Science, Technology & Research (A*STAR), Singapore, Singapore; 2 Cavendish Laboratory, University of Cambridge, Cambridge, UK; 3 Institute of Materials Research & Engineering, Agency for Science, Technology & Research (A*STAR), Singapore, Singapore; 4 Theoretical Division and Center for Nonlinear Studies, Los Alamos National Laboratory, Los Alamos, NM USA; 5 Physics Department, National University of Singapore, Singapore, Singapore

**Keywords:** Spintronics, Magnetic devices

## Abstract

Magnetic skyrmions are nanoscale spin textures touted as next-generation computing elements. When subjected to lateral currents, skyrmions move at considerable speeds. Their topological charge results in an additional transverse deflection known as the skyrmion Hall effect (SkHE). While promising, their dynamic phenomenology with current, skyrmion size, geometric effects and disorder remain to be established. Here we report on the ensemble dynamics of individual skyrmions forming dense arrays in Pt/Co/MgO wires by examining over 20,000 instances of motion across currents and fields. The skyrmion speed reaches 24 m/s in the plastic flow regime and is surprisingly robust to positional and size variations. Meanwhile, the SkHE saturates at ∼22^∘^, is substantially reshaped by the wire edge, and crucially increases weakly with skyrmion size. Particle model simulations suggest that the SkHE size dependence — contrary to analytical predictions — arises from the interplay of intrinsic and pinning-driven effects. These results establish a robust framework to harness SkHE and achieve high-throughput skyrmion motion in wire devices.

## Abstract (web summary)

Skyrmions - nanoscale, topological spin textures - are promising elements for next-generation computing due to their efficient coupling to currents in racetrack devices. Here, Tan et al. examine over 20,000 instances of current induced skyrmion motion to unveil a comprehensive picture of skyrmion dynamics across currents and fields.

## Introduction

Magnetic skyrmions are nanoscale, topologically wound spin structures stabilized in a ferromagnetic background by competing magnetic interactions^1,2^. Their discovery at room temperature (RT) in chiral multilayer films comprising heavy metal-ferromagnet interfaces has sparked scientific and technological excitement^3–6^. Notably, the spin-orbit torque (SOT) generated at such interfaces by an in-plane charge current^7,8^ provides the ideal instrument for electrical manipulation of skyrmions^5,9,10^. Consequently, several device proposals seek to harness current-driven skyrmion motion within a wire, or ‘racetrack’ architecture^11,12^ towards applications in analog memory^13^, logic^14^, and synaptic computing^15,16^. In this light, achieving deterministic, efficient, and high-throughput skyrmion motion in wires is a critical challenge for the device community.

Initial demonstrations of skyrmion motion were extremely promising — showing efficient SOT manipulation^10^ and individual speeds of up to ~100 m/s^5^. However, progress since has been limited by significant challenges along three key fronts pertaining to the influence of intrinsic, extrinsic, and collective effects. First, in addition to the expected linear motion, an applied current also induces a traverse skyrmion deflection — known as the skyrmion Hall effect (SkHE) — which arises from the hydrodynamic Magnus force acting on the skyrmionic topological charge^1,17–19^. SkHE may enable defect avoidance^20^, however the transverse deflection — over 30^∘^ in some cases — may limit linear mobility^18,21,22^. While efforts to develop SkHE-free materials are underway^23–27^, several facets of SkHE remain unresolved amidst conflicting experimental results, e.g. the material-dependence of its magnitude, the existence of a saturation value, dependence on skyrmion size, etc.^17,18,22,23,28^. Next, skyrmion dynamics in sputtered multilayer wires may be affected on one hand by material granularity and defects^29,30^, and on the other by interactions with the wire edge^31,32^. However, these extrinsic effects are yet to be experimentally understood. Finally, high-throughput devices would require skyrmion motion at densities 10–100 times higher than prevailing experiments^11^, which have examined sparse configurations (< 1 μm^−2^).

Here we report on the ensemble dynamics of 80–200 nm-sized skyrmions forming dense (> 10 μm^−2^) arrays in Pt/Co/MgO multilayer wires. Using magnetic force microscopy (MFM) imaging, we examine over 20,000 instances of skyrmion motion over a range of applied currents and fields, spanning three distinct dynamic regimes: stochastic creep, deterministic creep and plastic flow. The onset of the deterministic motion is associated with finite SkHE, which grows and saturates at a moderate value (~22^∘^). While the velocity is found to be surprisingly robust to edge effects and skyrmion size variations, the SkHE is considerably reshaped in both cases. Our simulations suggest that the observed SkHE trend with skyrmion size — contrary to defect-free theoretical predictions — arises from the interplay of intrinsic and pinning-driven effects. Our results and insights establish a robust experimental framework to realize high-throughput skyrmion motion in wire devices for next-generation nanoelectronics.

## Results

### Imaging skyrmion dynamics

**Fig. 1:**
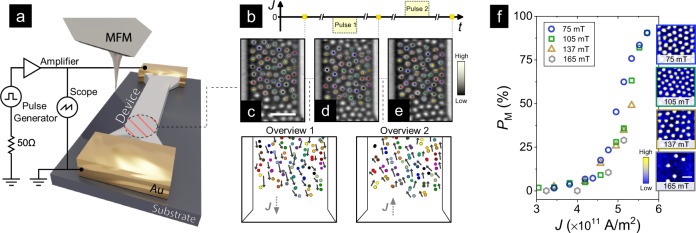
Experimental setup and device characterization. **a** Schematic of the experimental setup. The [Pt(3)/Co(1.2)/MgO(1.5)]_15_ (thicknesses in nm in parentheses) was mounted onto the MFM setup with varying *in situ* out-of-plane (OP) magnetic fields (details in Methods). **b** Protocol used to inject current pulses (magnitude *J* = ±(1.0−5.8) × 10^11^ A/m^2^, width: 20 ns) into the device. Pulses of alternating polarity were applied sequentially, and the wire was imaged by *in situ* MFM before and after each pulse. **c**–**e** Representative MFM images (scalebar: 1 μm) acquired at OP magnetic fields *μ*_0_*H* = 105 mT before pulsing (**c**), after applying current pulses of *J* ≃ ∓ 5.7 × 10^11^ A/m^2^ (**d**, **e**). Bottom left (right) panel shows tracked positions of selected skyrmions identified by colored dots for −*J* (**c**, **d**) and +*J* (**d**, **e**) (details in SM4), whose motion is indicated by arrows. **f** Proportion, *P*_M_, of skyrmions in motion along the driving current direction plotted as a function of *J* for various OP fields. Insets show MFM images (scale bar: 500 nm) of the wire at varying *μ*_0_*H* (magnitudes in insets) following the skyrmion nucleation recipe (see Methods). Finite *P*_M_ indicates the onset of deterministic skyrmion motion.

This work was performed at RT on [Pt(3)/Co(1.2)/MgO(1.5)]_15_ multilayer films sputtered on Si/SiO_2_ substrates and patterned into 2 μm wide wire devices (thickness in nm in parentheses, see Methods, SM1-2). The wires were connected through a circuit board to a pulse generator (Fig. 1a) to inject current pulses of fixed width (20 ns), varying magnitude and polarity, *J* = ±(1.0−5.8) × 10^11^ A/m^2^. The setup was mounted in an MFM with out-of-plane (OP) magnetic field (*μ*_0_*H*), enabling sequential *in situ* pulse injection and imaging of the wire (Fig. 1a, b). Pt/Co/MgO stacks are known to host Néel-textured skyrmions stabilized at RT by the interfacial Dzyaloshinskii-Moriya interaction^3,22^. Following an established nucleation recipe (see Methods, SM3), skyrmion configurations were stabilized in the wires over a substantial field range — *μ*_0_*H* ~ 75–165 mT — as seen in MFM images (Fig. 1f: inset). The range of skyrmion densities (*n*_S_: 2–13 μm^−2^) and sizes (*d*_S_: 80–200 nm) achieved here (details in SM3) are vastly different from previous works^5,17,18,21–23,28,30^, and provide the variance required to establish statistical significance for our key claims.

Current pulses of alternating polarity were applied sequentially to the wire (Fig. 1b) and the motion of individual skyrmions was quantified by tracking their positions from MFM images acquired before and after each pulse (Fig. 1c–e, details in SM4). Figure 1f summarizes the proportion of skyrmions, *P*_M_, moving along the current direction (*J*) for various fields. We expect *P*_M_ to be positive for current-induced SOT displacement of left-handed Néel skyrmions stabilized in Pt/Co-based/MgO stacks^3,5,33^. For *J* < 4 × 10^11^ A/m^2^, *P*_M_ remains below 5% — characteristic of stochastic motion due to current-induced thermal fluctuations^30,34^. As *J* is further increased, *P*_M_ increases exponentially — reaching ~90% at *J* ≃ 5.8 × 10^11^ A/m^2^ — indicating a transition to driven skyrmion motion along the current direction. Finally, such deterministic motion can be further demarcated as creep or flow, which is discussed further below.

### Skyrmion Hall effect

**Fig. 2:**
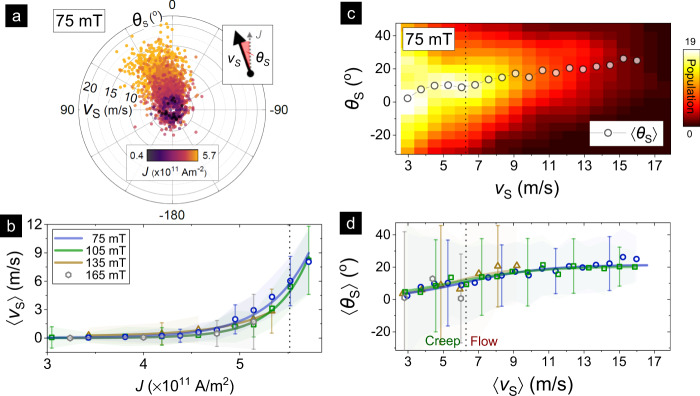
Current-driven skyrmion dynamics overview. **a** Polar plot overview of the skyrmion motion statistics for *μ*_0_*H* ≃ 75 mT across all currents *J* (data for *J* < 0 flipped by 180^∘^), showing the spread of velocity (*v*_S_) and angular deflection (*θ*_S_). The data is visibly biased towards the upper left quadrant. Inset shows a schematic defining *v*_S_ and *θ*_S_ relative to the direction of *J*. **b** Plot of the average skyrmion speed, 〈*v*_S_〉, against *J* for various applied fields. Solid lines present exponential fits (see Methods), while shaded regions represent the standard deviation, also emphasized by error bars for selected points. The dotted line — determined from (**d**) — demarcates creep and plastic flow regimes. **c** 2D histogram color plot of skyrmion deflection, *θ*_S_ against *v*_S_ for *μ*_0_*H* ≃ 75 mT across all currents. The data were binned by *v*_S_ for this plot. Solid markers show the average deflection, 〈*θ*_S_〉, for each *v*_S_ bin. **d** Plot of 〈*θ*_S_〉 against 〈*v*_S_〉 — determined as in (**c**) — for various applied fields. Solid lines present sigmoidal fits (see Methods), while shaded regions represent the standard deviation, also emphasized by error bars for selected points. Creep and plastic flow regimes are demarcated at $\langle {\theta }_{{\rm{S}}}\rangle =0.5\ {\langle {\theta }_{{\rm{S}}}\rangle }^{{\rm{sat}}}$ using the fit.

The polar plot in Fig. 2a summarizes the distribution of skyrmion dynamics across applied currents at a representative field (*μ*_0_*H* ≃ 75 mT). The variance in skyrmion velocity (*v*_S_) and angular deflection (*θ*_S_) — both defined with respect to *J* (Fig. 2a: inset) — is expected due to the granularity inherent to metallic multilayers^29,30^. Nevertheless, it is established that adequate statistical sampling and controls can meaningfully describe the skyrmion dynamics phenomenology^28,29^. Correspondingly, Fig. 2b shows a plot of the averaged skyrmion velocity 〈*v*_S_〉 for each *J*. Notably, 〈*v*_S_〉 increases exponentially with *J*, reaching ~8 m/s for *J* ≃ 5.8 × 10^11^ A/m^2^, with some skyrmions moving at over 24 m/s. The velocities observed here are of the same order of magnitude as Co-based skyrmionic counterparts subjected to similar *J*^5,21,22^. Next, the threshold current for skyrmion motion increases with applied field, which can be ascribed to stronger pinning effects^29,30^. Importantly, however, the exponential trend of 〈*v*_S_〉(*J*) is field-independent, indicating its insensitivity to variations in *n*_S_ for a given material system (c.f. Fig. 1f, see SM3). However, with increasing field, the viable range of currents is substantially reduced (e.g. *J* < 5.2 × 10^11^ A/m^2^ for *μ*_0_*H* ≃ 165 mT) due to the increased ease of skyrmion annihilation and stronger pinning^5,30^. Therefore, after demonstrating the consistency of skyrmion dynamics across fields (Figs. 1 and 2), the remainder of our work focuses — without loss of generality — on lower fields (75–105 mT) for statistically meaningful conclusions.

Turning now to the angular deflection, skyrmion motion in Fig. 2a is noticeably skewed towards the upper left quadrant (*θ*_S_ > 0) — increasingly so at higher velocities. The observed deflection is consistent with the expected SkHE, as skyrmions with topological charge *Q* = +1 should possess a positive intrinsic Hall angle (*θ*_H_ > 0)^1^. The 2D histogram plot in Fig. 2c shows the variation of 〈*θ*_S_〉 with 〈*v*_S_〉 across currents. For lower values of 〈*v*_S_〉 e.g. ≲6 m/s, 〈*θ*_S_〉 is small and monotonically increasing while displaying a large variance. These attributes are characteristic of creep motion in a disordered background — wherein scattering from the large fraction of pinned skyrmions results in a wide spread in 〈*θ*_S_〉^19,28,30^. As 〈*v*_S_〉 increases further, 〈*θ*_S_〉 narrows in spread and grows in an S-curve fashion — eventually saturating at ${\theta }_{{\rm{S}}}^{\rm{sat}} \sim 2{2}^{\circ }$ for 〈*v*_S_〉 ≳ 12 m/s. The 〈*θ*_S_〉 saturation signals the onset of plastic flow, wherein skyrmions move concomitantly (*P*_M_ > 50%) in a weak pinning background^19,22,28^.

As shown in Fig. 2d, both the 〈*θ*_S_〉(*v*_S_) profile and ${\theta }_{{\rm{S}}}^{\mathrm{sat}}$ magnitude are consistent over the entire field range (75–165 mT). This enables us to clearly demarcate the creep and plastic flow regimes in our work (Fig. 2d), which in turn establishes the current regime corresponding to plastic skyrmion flow (Fig. 2c: *J* ≥ 5.5 × 10^11^ A/m^2^). Such a demarcation, while contrasting with some reports that solely exhibit either creep^18^ or plastic flow^28^, is consistent with other works^17,22,23^. Surprisingly, the saturated magnitude of ${\theta }_{{\rm{S}}}^{\mathrm{sat}} \sim 2{2}^{\circ }$ is nearly 2–3 times lower than the 40–70^∘^ range found in most ferromagnetic multilayers^17,18,22^, and is comparable to the 25–35^∘^ values for ferrimagnetic systems^23,25^. Crucially, the established robustness with field enables us to examine extrinsic influences on the plastic flow of individual skyrmions in subsequent sections.

### Effect of wire edge

**Fig. 3:**
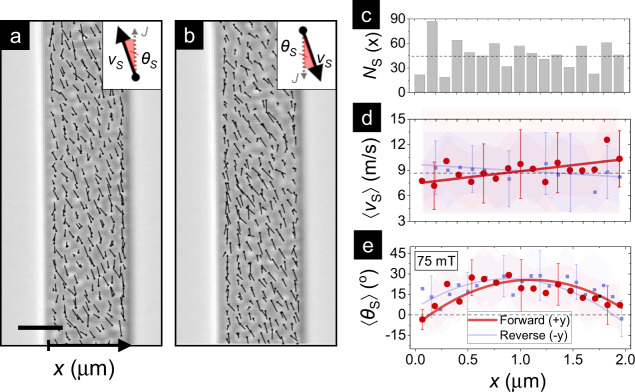
Confinement effects on skyrmion flow dynamics. **a**, **b** Representative MFM images (scale bar: 1 μm) at *μ*_0_*H* ≃ 75 mT showing the forward ($J\parallel +\hat{y}$) (**a**) and reverse ($J\parallel -\hat{y}$) (**b**) plastic flow of skyrmions with varying distance *x* from the left edge. Overlaid dots correspond to initial skyrmion positions and lines show the extent of motion due to the current pulse. Insets define *v*_S_ and *θ*_S_ with respect to *J* for both cases. **c** Binned histogram distribution of skyrmions based on their *x*-positions before motion. **d**, **e** Average velocity $\left\langle {v}_{{\rm{S}}}(x)\right\rangle$ (**d**) and angular deflection $\left\langle {\theta }_{{\rm{S}}}(x)\right\rangle$ (**e**) for skyrmions in each *x*-bin for forward (*J*∥+$\hat{y}$, red) and reverse (*J*∥-$\hat{y}$, blue) motion (details in SM7). Solid lines serve as guides-to-the-eye to indicate trends, while shaded regions represent the standard deviation, also emphasized by error bars for selected points.

Geometric confinement can strongly influence the stability and dynamics of skyrmions via magnetostatic and torque contributions^9,13,35^. Previous theoretical and experimental works on single skyrmion in the creep regime have studied their interaction with a geometric boundary or “edge”, and variously reported edge-induced skyrmion pinning^17,36^, annihilation^31,36,37^, expulsion^13,32,37^ or repulsion^13,17,31^. Here we examine the influence of confinement on the plastic flow of skyrmion arrays. Skyrmions in the plastic flow regime are binned by their individual distance *x* from the left edge of the wire, with 0 < *x* < 2 μm (see SM7). The binned skyrmion number *N*_S_(*x*) (Fig. 3c) is approximately uniform across the *x*-bins, with no evidence for preferential existence at the center or edge. Meanwhile Fig. 3a, b show a marked evolution in the dynamics with varying *x*-positions, which is quantified in Fig. 3d, e.

Figure 3d shows that the binned velocity 〈*v*_S_(*x*)〉 remains consistently high (~8–10 m/s) across the wire width. A slight (~20%) decrease of 〈*v*_S_(*x*)〉 is found at the left edge of the wire — i.e. in the direction of skyrmion deflection. This suggests that skyrmion-edge interaction may have a measurable inelastic component — consistent with a dissipative process^17,31^. Meanwhile, the binned deflection 〈*θ*_S_(*x*)〉 shows a parabolic evolution across the wire: increasing from ~−5^∘^ at the left to ~+25^∘^ at the center (c.f. Fig. 2d, ${\theta }_{{\rm{S}}}^{\mathrm{sat}} \sim 2{2}^{\circ }$) and then dropping to ~+10^∘^ at the right. Crucially, we observe the same evolution of 〈*v*_S_(*x*)〉 and 〈*θ*_S_(*x*)〉 when the motion is reversed (albeit with flipped sign: see Fig. 3d, e) and at higher fields (105 mT, see SM6). Noting the consistency of edge effects across field variations of 30 mT, we rule out the role of current-induced Oersted fields in the observed trends.

Instead, the 〈*θ*_S_(*x*)〉 variation may be interpreted within the interplay of the intrinsic Hall effect of individual skyrmions with extrinsic effects from the wire edge and neighboring skyrmions. First, near the center (*x* ~ 1 μm), edge effects are negligible and neighboring skyrmion effects are compensated on both sides. Therefore, the ensuing 〈*θ*_S_〉 is comparable to the saturation value (see Results: Skyrmion Hall effect). Next, at the left edge (*x* ~ 0 μm), the Magnus force is overcome by edge repulsion. The latter pushes the skyrmion back into the wire — resulting in a negative 〈*θ*_S_〉. Finally, the gradual reduction of 〈*θ*_S_〉 for (1 < *x* < 2 μm) cannot be explained by edge effects. Instead, it suggests that the gradual reduction of skyrmions on the right — whose transverse motion may repel the skyrmion of interest — may influence the magnitude of 〈*θ*_S_〉 observed in our work. Finally, the robustness of these results to field and current direction underscores the deterministic role of extrinsic factors in shaping skyrmion dynamics in our wires.

### Effect of skyrmion size

**Fig. 4:**
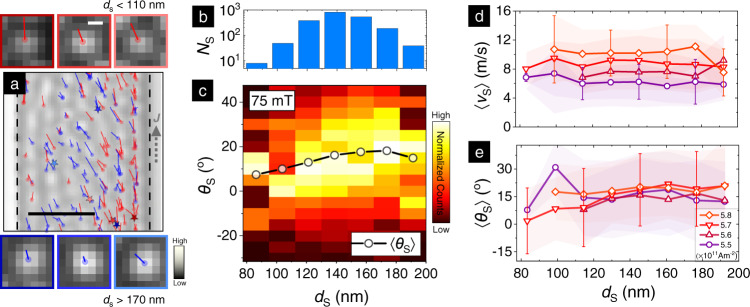
Skyrmion size effect on flow dynamics. **a** Representative cropped MFM image (scale bar: 1 μm) at *μ*_0_*H* ≃ 75 mT showing the evolution in plastic flow of skyrmions with varying size *d*_S_. The smallest 25% (red) and largest 25% (blue) of *d*_S_ are highlighted. Overlaid dots correspond to initial skyrmion positions and lines show the extent of motion. Zoomed insets at top and bottom (scalebar: 100 nm) show selected small (top, < 110 nm) and large (bottom, >170 nm) skyrmions for comparison. **b** Binned histogram distribution of skyrmions, *N*_S_, based on their size, *d*_S_—which varies over 80–200 nm (details in SM7). **c** 2D histogram color plot of skyrmion deflection, *θ*_S_ against *d*_S_ for *μ*_0_*H* = 75 mT across all currents in the plastic flow regime. The data were binned by *d*_S_ and normalized in each bin for this plot. Solid markers show the average deflection, 〈*θ*_S_〉, for each *d*_S_ bin. Average velocity 〈*v*_S_〉 (**d**) and angular deflection 〈*θ*_S_〉 (**e**) for skyrmions in each *d*_S_-bin for *J* over (5.5–5.8) × 10^11^ A/m^2^. Shaded regions represent the standard deviation, also emphasized by error bars for selected points.

The size of a skyrmion determines its coupling to current-induced spin torques, and is therefore expected to influence its dynamics^38,39^. In Fig. 4, we examine the skyrmion size dependence seen in our experiments in the plastic flow regime for *μ*_0_*H* ≃ 75 mT and *J* over (5.5–5.8) × 10^11^ A/m^2^. The skyrmions are binned by their *d*_S_ in MFM images (see SM7), which show a substantial spread over 80–200 nm (Fig. 4a insets, Fig. 4b). First, Fig. 4d shows that 〈*v*_S_〉 — while increasing as expected with *J* — is constant to ~10% across *d*_S_ for fixed *J*. Such insensitivity of 〈*v*_S_〉 to *d*_S_, predicted for *d*_S_ > 100 nm and recently reported for 〈*d*_S_〉 ~ 400 nm skyrmion bubbles^28^, may be ascribed to the onset of finite size effects within the skyrmion spin structure^30^.

Meanwhile, the variation of 〈*θ*_S_〉 with *d*_S_ across currents is shown in the 2D histogram plot in Fig. 4c. 〈*θ*_S_〉 increases discernibly with *d*_S_ across all *J*-values in the plastic flow regime (Fig. 4e) — from ~5–10^∘^ for *d*_S_ ≲ 100 nm to ~20^∘^ for *d*_S_ ~ 200 nm. While the limited current range for plastic flow (5.5–5.8 × 10^11^ A/m^2^) precludes meaningful *J*-dependent trends, the weakly increasing trend of 〈*θ*_S_〉 with *d*_S_ noted here is robust to binning (SM7). Moreover, similar results are observed for 105 mT (SM6, 7), and another wire device as well (SM6). Our results contrast strongly with the 1/*d*_S_ dependence of *θ*_H_ expected theoretically from the Thiele model for rigid skyrmions^38,40^, and with experimental reports of such a 〈*θ*_S_〉 trend in the creep regime^18^. Meanwhile, a recent work has reported *d*_S_-independent 〈*θ*_S_〉 in the plastic flow regime^28^. In light of contrasting theoretical and experimental reports, the size dependence of SkHE has increasingly assumed importance. Our simulations and theoretical work attempt to identify plausible origins of these discrepancies.

### Micromagnetics

**Fig. 5:**
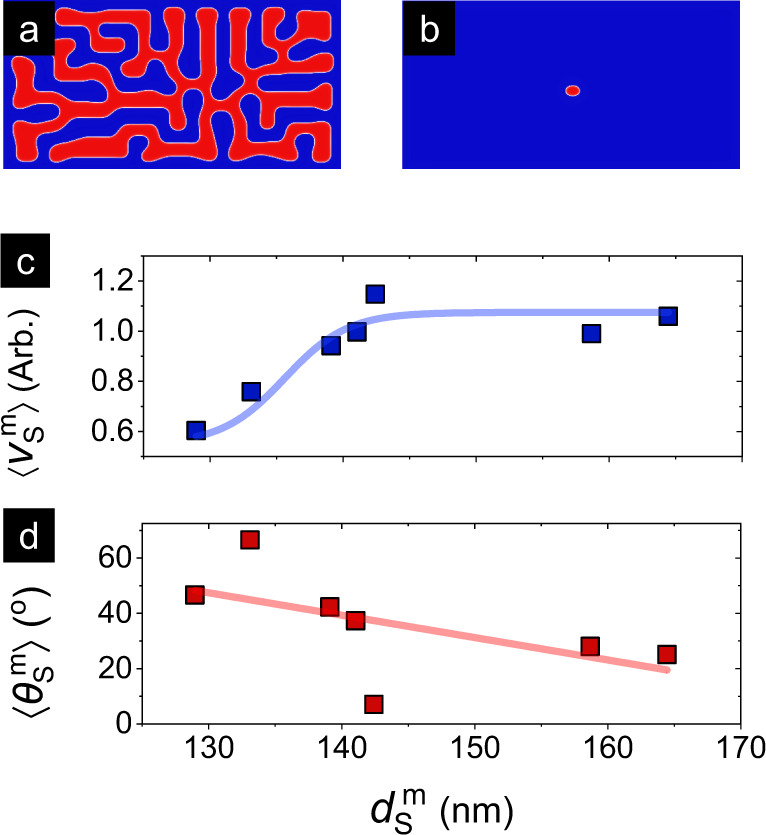
Micromagnetic simulations of skyrmion size effects. **a**, **b** Representative micromagnetic magnetization of a 2 × 4 μm^2^ wire showing a single skyrmion formed upon relaxation at *μ*_0_*H* ≃ 85 mT. The grain-free simulations were performed using stack parameters consistent with experiments (details in Methods). **c**, **d** Evolution of micromagnetic simulated average velocity $\langle {v}_{{\rm{S}}}^{{\rm{m}}}\rangle$ (**c**) and average angular deflection $\langle {\theta }_{{\rm{S}}}^{{\rm{m}}}\rangle$ (**d**) with *d*_S_ — extracted from a series of such simulations at a representative *J* = 9.5 × 10^11^ A/m^2^. Solid lines represent sigmoidal (**c**) and linear (**d**) fits, respectively.

Micromagnetic simulations were performed for a grain-free environment using stack parameters consistent with experiments to study the size dependence of the intrinsic SkHE (see Methods, SM8)^41^. Skyrmions were stabilized in a 2 × 4 μm wire geometry with varying *μ*_0_*H* (84–93 mT, see e.g. Fig. 5a, b), which resulted in a 20% variation in simulated *d*_S_ over 129–164 nm. Analysis of the current-induced skyrmion motion, shown in Fig. 5c, d for *J* = 9.5 × 10^11^ A/m^2^, reveals that with increasing ${d}_{{\rm{S}}}^{{\rm{m}}}$, the simulated $\langle {v}_{{\rm{S}}}^{{\rm{m}}}\rangle$ initially increases and eventually saturates for *d*_S_ ≳ 150 nm. Meanwhile $\langle {\theta }_{{\rm{S}}}^{{\rm{m}}}\rangle$ decreases from 40^∘^ to 20^∘^. Both these trends are consistent with the expected rigid skyrmion behavior and with recent simulations in the creep regime^18,30^. However, neither our grain-free micromagnetic simulations nor those incorporating inhomogeneity^22,29,30^ can explain the *d*_S_-dependent $\langle {\theta }_{{\rm{S}}}^{\rm{m}}\rangle$ trends in the plastic flow regime seen in Fig. 4, or in other recent works^28^. Therefore, we turn to the particle model — an established technique for elucidating the dynamics of skyrmion arrays in a disordered background (see Methods)^19,42^.

### Particle model

**Fig. 6:**
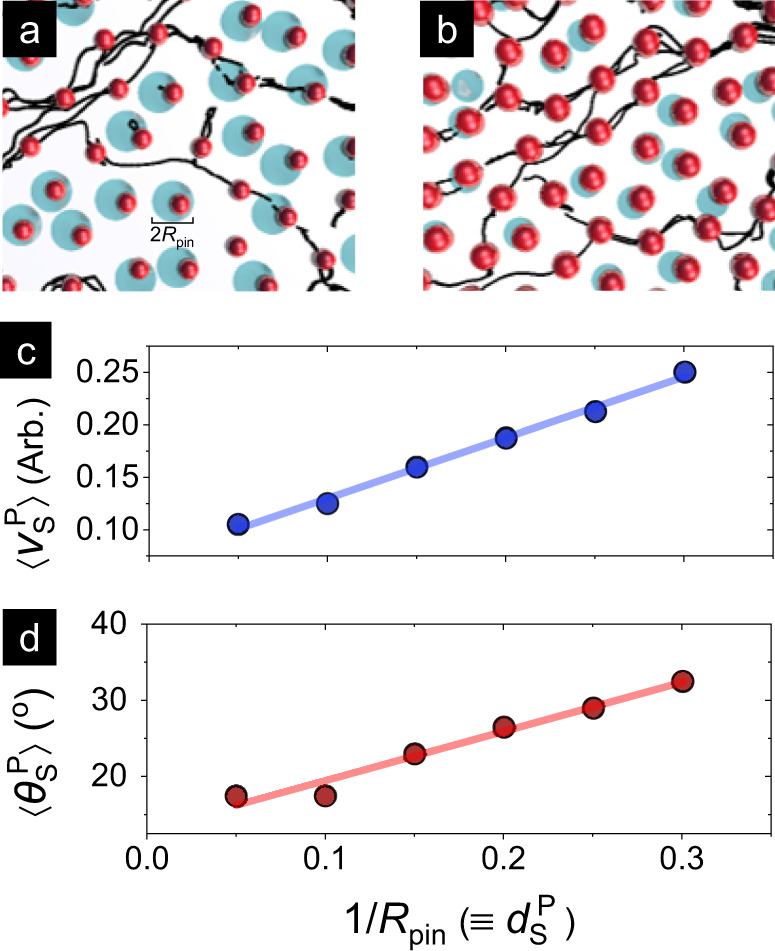
Particle model simulations of skyrmion size effects. **a**, **b** Schematics of particle model simulations (details in Methods) which examine the dynamics of point-like skyrmion particles (foreground, red) in the presence of pinning sites with radius *R*_pin_ (background, blue). Black lines show representative trajectories of skyrmions. Decreasing *R*_pin_ within the model (left to right) emulates the effect of increasing *d*_S_. **c**, **d** Evolution of particle model simulated average velocity $\langle {v}_{{\rm{S}}}^{{\rm{p}}}\rangle$ (**c**) and average angular deflection $\langle {\theta }_{{\rm{S}}}^{{\rm{p}}}\rangle$ (**d**) with 1/*R*_pin_ ≡ *d*_S_ extracted from a series of particle model simulations with fixed drive.

As shown in Fig. 6a, b, skyrmions are represented as an array of point particles, while the disorder is modeled using pinning sites of radius, *R*_pin_ (details in Methods). Within this model, the effectiveness of pinning scales with the area coverage, given by ${({d}_{{\rm{S}}})}^{2}/{({R}_{{\rm{pin}}})}^{2}$, where *d*_S_ is the skyrmion size. Therefore, the impact of changing skyrmion size can be modeled either by increasing/decreasing *d*_S_ or by increasing/decreasing 1/*R*_pin_. Here, we increase *R*_pin_ to emulate the effect of reducing *d*_S_ (Fig. 6a), wherein smaller skyrmions would experience a larger interaction landscape with a given pinning site. The ${\rm{d}}_{{\rm{S}}}^{{\rm{P}}}$ in Fig. 6c, d is described as a dimensionless number based on the ratio of the *d*_S_ to *R*_pin_. The simulations were seeded with 1500 particles and 1200 pinning sites, and the system was subjected to a fixed drive corresponding to plastic flow under the modified Thiele equation (see Methods). The intrinsic, or pin-free skyrmion Hall angle, *θ*_H_, was set to 37^∘^, while *R*_pin_ was varied over a wide range to simulate the role of pinning effects in the observed size dependence of skyrmion dynamics.

On one hand, Fig. 6d shows that the particle model angular deflection, $\langle {\theta }_{{\rm{S}}}^{{\rm{p}}}\rangle$ decreases monotonically with reducing *d*_S_ (simulated by increasing *R*_pin_) to values substantially below the intrinsic Hall angle (*θ*_H_ = 37^∘^). Additional simulations for *θ*_H_ = 45^∘^ confirm this trend, which is qualitatively consistent with experiments. The simulations suggest that while smaller skyrmions may in fact have a higher intrinsic *θ*_H_, as per the Thiele model^38,40^, extrinsic interactions may considerably reshape the effective $\langle {\theta }_{{\rm{S}}}^{{\rm{p}}}\rangle$. In particular, the stronger influence of pinning on smaller skyrmions may impede their transverse deflection, reducing $\langle {\theta }_{{\rm{S}}}^{{\rm{p}}}\rangle$ to well below the intrinsic value. Meanwhile the simulation results also suggest the *d*_S_-independent SkHE reported in another recent work^28^ may result from the dominance of pinning effects over the intrinsic Hall effect. In this case, the effective pinning could be acting at scales much larger than *d*_S_, and could therefore result in the diminished contribution of variations in *d*_S_. On the other hand, Fig. 6c shows that the particle model velocity, $\langle {v}_{{\rm{S}}}^{{\rm{p}}}\rangle$ increases linearly with 1/*R*_pin_ ≡ *d*_S_, likely because increased pinning would slow down point-like skyrmions. In comparison, the *d*_S_-independence of *v*_S_ in our experiments and previous work^28^ may be attributed, from micromagnetic simulations, to the onset of bubble-like internal structure of skyrmions^30^. Such textural size effects, whose internal modes may, for smaller *d*_S_, also reduce the effective magnitude of adiabatic and non-adiabatic damping, are beyond the scope of these particle model simulations. Future theoretical works may incorporate these effects and consider the skyrmion interactions arising from the vastly higher magnitude of skyrmion densities in our experiments. Nevertheless, these insights provide an important stepping stone for delineating intrinsic and extrinsic contributions to skyrmion dynamics.

## Discussion

In summary, we have presented a systematic study of the ensemble dynamics of skyrmions forming dense array configurations in multilayer wire devices. With increasing current, we observe distinct transitions in skyrmion dynamics — from stochastic to deterministic creep, accompanied by SkHE onset, and then to plastic flow — wherein SkHE saturates at ~22^∘^. Within the plastic flow regime, we find that the skyrmion velocity is surprisingly robust to edge effects and skyrmion size, while the SkHE varies considerably in response to both. Notably, the weak increase of SkHE with skyrmion size contradicts existing predictions for isolated, rigid skyrmions. Instead, we conclude that the intrinsic Hall effect of skyrmions is strongly reshaped by extrinsic contributions in our wires — including geometric confinement, disorder-induced pinning effects and skyrmion-skyrmion interactions.

The insights from our work dispel several prevailing notions on skyrmion dynamics in multilayers, and pave imminent materials and device directions for high-throughput racetrack devices. First, the relatively small magnitude of SkHE in our case (saturated, ~22^∘^) is comparable to ferrimagnetic multilayers^23^. This suggests that materials design efforts for optimizing SkHE should continue exploring conventional ferromagnets in addition to newer compensated systems. Second, we find the skyrmion-edge interaction to be weakly inelastic: unlike several predictions^32,36,37^, the edge does not annihilate or pin skyrmions. In fact, such skyrmion-edge interactions may be exploited to channel skyrmion motion within suitably designed device geometries^14^. Third, size effects on skyrmion dynamics are reshaped by material granularity and skyrmion-skyrmion interactions. Contrary to defect-free theoretical predictions, in our case smaller skyrmions move equally fast, and importantly with reduced SkHE. While emphasizing accurate inclusion of these effects in future theoretical works, we postulate that they may serve as tuning parameters to achieve bespoke size-dependent skyrmion dynamics in racetrack devices^43,44^. Finally, we note that the striking individuality of our skyrmions within dense, disordered arrays bodes well for their use as stochastic spiking neurons in synaptic computing applications^15,16^.

## Methods

### Film deposition

Multilayer stacks of Ta(3)/[Pt(3)/**Co(1.2)**/MgO(1.5)]_15_/Ta(4) (nominal layer thicknesses in nm in parentheses) were deposited on pre-cleaned thermally oxidized 200 mm Si wafers by ultrahigh vacuum magnetron sputtering at RT using the Singulus Timaris^TM^ system. Magnetization measurements were performed on the film using the MicroSense^TM^ Model EZ11 vibrating sample magnetometer. An effective OP anisotropy, *K*_eff_ of 0.17 MJ/m^3^ and saturation magnetization, *M*_s_ of 1.1 MA/m were determined from the *M*(*H*) data. The DMI, *D* and exchange stiffness, *A* of the film were determined to be 1.6 mJ/m^2^ and 24 pJ/m, respectively, using established techniques^4–6^. These values are in line with published results on Pt/Co(0.9)/MgO stacks^3,22^.

### Device fabrication

A 300 nm thick negative resist, Ma-N 2403, was spin-coated on the multilayer film. Wires of dimensions 2 × 10 μm were exposed using the Elionix^TM^ electron beam lithography tool. The patterns were transferred onto the multilayer film using an Oxford CAIBE^TM^ ion beam etching system, and residual resist was lifted off in an ultrasonic bath. Top electrodes were subsequently patterned using an EVG^TM^ optical mask aligner, followed by the deposition of the electrode stack Ta(5)/Au(100)/Ru(20) using the Chiron^TM^ UHV magnetron sputtering system.

### MFM and electrical pulsing setup

MFM imaging was performed using a Veeco Dimension^TM^ 3100 scanning probe microscope with Co-alloy coated SSS-MFMR^TM^ tips. The sharp tip profile (diameter ~30 nm), ultra-low moment (~80 emu/cm^3^), and lift heights of 20–30 nm used during scanning provided high-resolution MFM images while introducing minimal stray field perturbations. Our earlier works have established MFM as a reliable tool for imaging sub-100 nm skyrmions in multilayer films^6,45^. *In situ* electrical pulsing and imaging were carried out in our custom-designed platform consisting of a Tektronix^TM^ AFG3252 pulse generator, SRS^TM^ SIM954 inverting amplifier, Tektronix^TM^ TDS 7404 oscilloscope and the microscope. The device under test was wire-bonded to the chip carrier and subsequently mounted onto the MFM setup with varying *in situ* OP magnetic fields of 75–165 mT, following *ex situ* negative OP saturation. The device was impedance matched and found to be ~50 Ω. Ambipolar pulses of amplitude 1–3.7 V were injected, corresponding to current densities of (1.0–5.8) × 10^11^ A/m^2^ assuming a total metallic layer thickness of 63 nm. Short pulse duration of 20 ns was used to limit Joule heating effects on skyrmion nucleation, deletion and motion. The resistance and zero field configuration of the devices were verified prior to and after electrical pulsing, affirming that the pristine device form was preserved over the course of electrical pulse injection experiments.

### Electrical pulsing experiments

Magnetic configurations consisting solely of skyrmions were stabilized over these fields by injecting bipolar current pulses of magnitude *J* < 5.5 × 10^11^ A/m^2^ (details in SM3). The procedure was repeated until all stripes were broken up into skyrmions, and no further skyrmions could be created. Subsequently, current-driven skyrmion dynamics experiments were performed with *J* ranging over (1.0–5.8) × 10^11^ A/m^2^. Skyrmion motion was analysed by identifying and tracking the skyrmion positions on the MFM image after each pulse. The 〈*v*_S_〉 and 〈*θ*_S_〉 were extracted by calculating their average displacement over an effective pulse duration of 20 ns, taking into account the rise and fall times. The 〈*v*_S_〉 vs *J* (Fig. 2b) and 〈*θ*_S_〉 vs 〈*v*_S_〉 (Fig. 2d) plots are fitted using the exponential (1) and sigmoidal (2) functions respectively, defined as

1 $$y=a+b\exp \left(\frac{x-c}{d}\right)$$

2 $$y=\frac{a}{1+\exp \left(-\frac{x+b}{c}\right)}+d$$

where *a*, *b*, *c* and *d* are constants.

### Skyrmion dynamics analysis

To ensure that the devices imaged after pulsing were at identical positions for reliable tracking and analysis of skyrmion motion, an image registration protocol established using the image processing toolbox in MATLAB^®^ was implemented. The MFM images obtained from the consecutive pulses were aligned by performing a 2D geometric transformation consisting of translation, rotation and shear relative to a reference image (described in SM4). Following image alignment, all skyrmions were identified and each was tagged with a unique marker. Next, each skyrmion was traced to its new position after pulsing from its original position by systematically tracking around its nearest position starting from top-down (or bottom-up, depending on the pulse direction). The rigorous tracking protocol accounts for nearly all skyrmion motion, as the local skyrmion number is typically unchanged through the pulsing experiments (see SM4).

### Micromagnetic simulations

Micromagnetic simulations were performed using the MuMax3 software^41^ on a rectangular area of 2 × 4 μm^2^ to mimic the experimental wire structure. In view of computational constraints, the 15 repeat stack was simulated with an effective medium model^5^, wherein each repeat was represented by one effective FM layer. The magnetic parameters used in the effective medium model were rescaled from the experimentally measured magnetic parameters, where *A* = 5.05 pJ/m, *M*_s_ = 0.23 MA/m, *K*_eff_ = 0.070 MJ/m^3^ and *D* = 0.37 mJ/m^2^ ^5^. An experimentally determined Gilbert damping parameter of *α* = 0.05 was used in this simulation (details in SM1). To simulate the skyrmion dynamics, the SOT on the Co layer was modeled as an anti-damping-like torque from the adjacent Pt layer with an effective spin-Hall angle of 0.1. For simplicity, field-like torque originating from Pt was not considered and simulations were carried out at zero-temperature. Additionally, the role of thermal heating was neglected in line with experimental observation of negligible current-induced heating effects (see SM3).

### Particle model simulations

Skyrmion dynamics within the particle model was simulated using a modified Thiele equation^19,42^

3 $${\alpha }_{{\rm{d}}}{v}_{i}+{\alpha }_{{\rm{m}}}\hat{Z}\times {v}_{i}={F}_{i}^{{\rm{ss}}}+{F}_{i}^{{\rm{sp}}}+{F}^{{\rm{D}}}$$

Here, *α*_d_ is the damping and *α*_m_ is the Magnus force. The term ${F}_{i}^{{\rm{ss}}}$ is the skyrmion repulsive interaction, and ${F}_{i}^{{\rm{sp}}}$ is the skyrmion-pinning interaction. The pinning was modeled as arising from localized sites of radius *R*_pin_ with a finite range harmonic attractive potential which gives a maximum pinning force strength of *F*_pin_. The term *F*^D^ represents a dc drive on the skyrmions applied in the *x*-direction. The skyrmion velocity parallel and perpendicular to the drive is ${v}_{{\rm{S}},\parallel }^{{\rm{P}}}$ and ${v}_{{\rm{S}},\perp }^{{\rm{P}}}$ respectively, while the measured skyrmion Hall angle is ${\theta }_{S}^{{\rm{P}}}={\tan }^{-1}({v}_{{\rm{S}},\perp }^{{\rm{P}}}/{v}_{{\rm{S}},\parallel }^{{\rm{P}}})$. In the absence of disorder, the skyrmion Hall angle is ${\tan }^{-1}({\alpha }_{{\rm{m}}}/{\alpha }_{{\rm{d}}})$. The simulations presented here were performed with the ratio of the number of pinning sites to the number of skyrmions being 0.6. To mimic the effect of changing the skyrmion diameter, the pinning force was held constant, and the pinning radius *R*_pin_ was varied. In this case, a large pinning site would correspond to a smaller skyrmion. The effective skyrmion Hall angle and the skyrmion velocity were then measured from the simulations. The values of *F*_pin_ = 1.0, *α*_d_ = 1.34, and *α*_m_ = 1.0 were used for simulations, giving an intrinsic Hall angle of 37^∘^.

## Supplementary information

Supplementary Information

## Data Availability

The data generated during and/or analysed during the current study are available from the corresponding author(s) on reasonable request.
