# Supplementary material for: Visualizing the strongly reshaped skyrmion Hall effect in multilayer wire devices
Source: Nat Commun. 2021 Jul 12;12:4252. doi: 10.1038/s41467-021-24114-8 (PMC8275747; doi:10.1038/s41467-021-24114-8)
Supplement: Supplementary file 1 — Supplementary Information [file 41467_2021_24114_MOESM1_ESM.pdf]

Supplementary Information for

Visualizing the Strongly Reshaped Skyrmion Hall Effect in Multilayer Wire Devices

Anthony K.C. Tan,[1,2,*] Pin Ho,[3,1,†] James Lourembam,[3,1] Lisen Huang,[3,1] Hang Khume Tan,[3,1] Cynthia J.O. Reichhardt,[4] Charles Reichhardt,[4] and Anjan Soumyanarayanan[3,1,5,‡]

[1] *Data Storage Institute, Agency for Science, Technology & Research (A*STAR), Singapore*

[2] *Cavendish Laboratory, University of Cambridge, Cambridge, UK*

[3] *Institute of Materials Research & Engineering, Agency for Science, Technology & Research (A*STAR), Singapore*

[4] *Theoretical Division and Center for Nonlinear Studies, Los Alamos National Laboratory, Los Alamos, NM, USA*

[5] *Physics Department, National University of Singapore, Singapore*

Contents

S1 Stack Structure & Magnetic Properties . . . 2

S2 Wire Device Fabrication . . . 3

S3 Skyrmion Nucleation & Properties . . . 4

S4 Skyrmion Motion Tracking Protocol . . . 6

S5 Quantifying Skyrmion Dynamics Parameters . . . 8

S6 Additional Data for Skyrmion Flow Dynamics . . . 10

S7 Binning Effects . . . 12

S8 Micromagnetic Simulations . . . 15

S9 Experimental Skyrmion Dynamics Characteristics in Published Works . . . 17

References . . . 18

S1. Stack Structure & Magnetic Properties

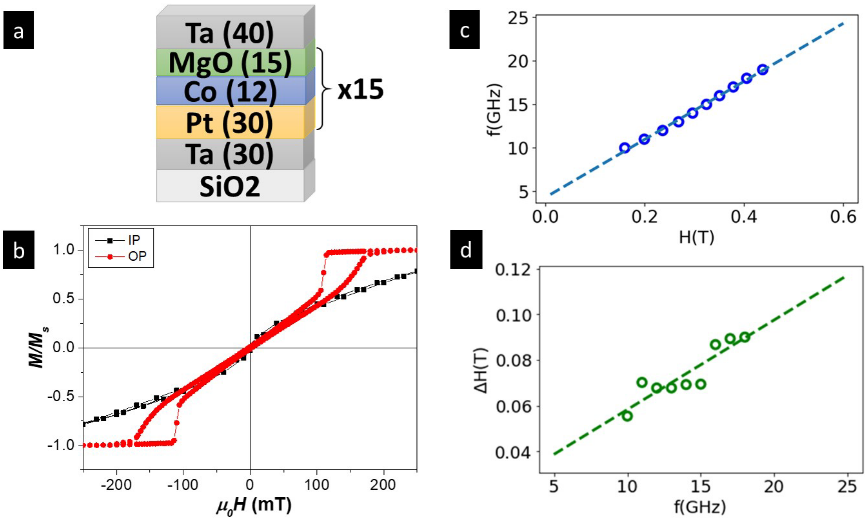

FIG. S1. **Stack Structure and Magnetic Parameters.** **(a)** Schematic representation of the multilayer stack containing 15 repeats of Pt(3)/Co(1.2)/MgO(1.5) (thickness in nm in parentheses). **(b)** Out-of-plane (OP, red) and in-plane (IP, black) magnetization hysteresis loops. **(c-d)** Fits to the ferromagnetic resonance (FMR) peak positions (c) and linewidths (d) for the determination of gyromagnetic ratio γ and Gilbert damping parameter α respectively.

Stack Structure. A multilayer film with 15 repeats of Pt(3)/Co(0.9-1.4)/MgO(1.5) (norminal thicknesses in nm in parentheses) was deposited on 8” thermally oxidized Si wafer using Singulus Timaris™. Coupon samples were precisely diced at positions of interest i.e. Co thickness of 1.2 nm, for subsequent characterization and device fabrication. The expected Co thickness variation along the wedge for a coupon size of 1×1 cm is negligibly small (< 0.02 nm). The complete stack structure, illustrated schematically in Fig. S1a consists of a Ta (3 nm) underlayer for adhesion and Pt texture and Ta (4 nm) cap for protection against oxidation.

Magnetic Parameters. The $[\mathrm{Pt}(3)/\mathrm{Co}(1.2)/\mathrm{MgO}(1.5)]_{15}$ multilayer thin film has an effective OP anisotropy (K_{eff}) of 0.17 MJ/m^3 and saturation magnetization (M_{s}) of 1.1 MA/m, as derived from out-of-plane (OP) and in-plane (IP) magnetic hysteresis measurements using vibrating sample magnetometry (Fig. S1b, details in Methods). The interfacial Dzyaloshinskii-Moriya interaction (DMI, D) of 1.6 mJ/m^2 and exchange stiffness (A) of 24 pJ/m were determined using an established combination of experiments and simulations. χ^2-fits were performed to the zero field domain periodicity (340 nm) measured by magnetic force microscopy (MFM) against micromagnetic simulation results for a wide range of D and A[1,2].

Gilbert Damping. The Gilbert damping parameter (α) was determined to be 0.05 from magnetization dynamics measurements using a home-built broadband vector network analyzer ferromagnetic resonance spectroscopy (VNA-FMR) setup[3,4]. The FMR spectra were obtained for external OP magnetic fields of up to 0.55 T over a frequency (f) range of 2 to 26 GHz, and fitted using Lorentz absorption and dispersion line-shape functions[4]. To obtain the gyromagnetic ratio γ (Fig. S1c), the FMR resonance peaks (H) were fitted using the Kittel equation for OP configuration, defined as[5]

$$f = \frac{\mu_0 \mid \gamma \mid}{2\pi}(H - M_{\mathrm{eff}}) \tag{S1}$$

where M_{eff} is the effective magnetization. The α (Fig. S1d) was then determined from the linear fit of the full width half maxima of the peaks (ΔH) vs f, defined as[3,6]

$$\Delta H = \frac{4\pi\alpha}{\mu_0 \mid \gamma \mid} f + \Delta H_0 \tag{S2}$$

where ΔH_0 represents the inhomogeneous linewidth broadening.

S2. Wire Device Fabrication

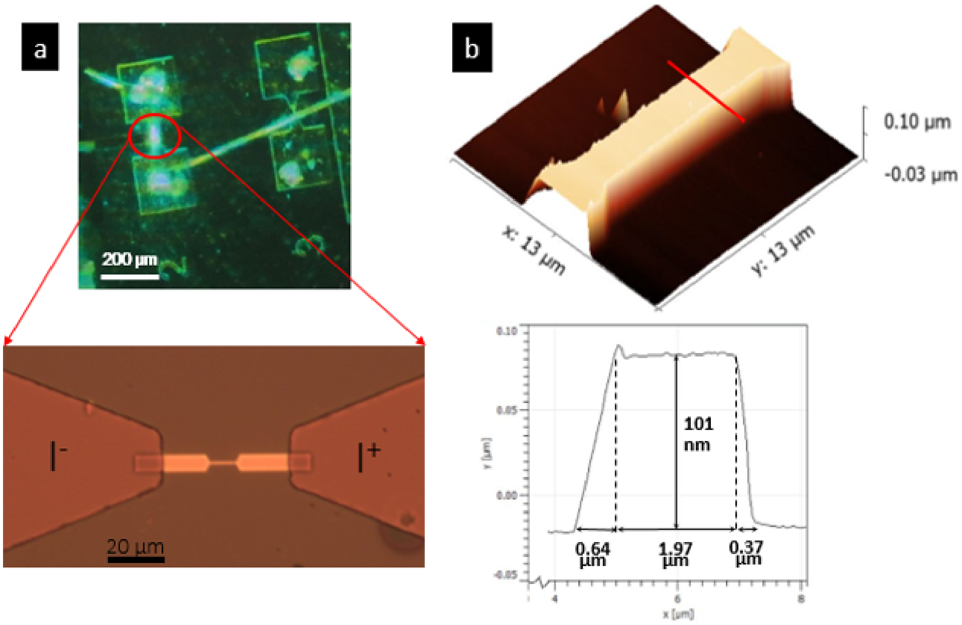

FIG. S2. **Wire Device Fabrication. (a)** Optical microscope image of a $[Pt(3)/Co(1.2)/MgO(1.5)]_{15}$ wire device with Ta(5)/Au(100)/Ru(20) (nominal layer thicknesses in nm in parentheses) electrode wire-bonded to the chip carrier. **(b)** Atomic force microscopy 3D image of a representative wire (top) and its cross-sectional height profile (bottom) at the wire position indicated in red.

Wire Profile. Coupon samples consisting of Pt(3)/Co(1.2)/MgO(1.5) multilayer films were patterned with electron beam lithography and etched, as detailed in Methods, to produce wire devices of dimensions $2 \times 10\,\mu$m (Fig. S2a). The Ta/Au/Ru electrodes were subsequently patterned on the wire (see Methods) for wire-bonding to the chip carrier. The etching process was closely monitored with an end-point detector and over-etched by $\sim$ 10 nm into the SiO_2. Atomic force microscopy of a representative wire (Fig. S2b) shows a tapered wire profile with intermittent defects along the edges due to incomplete lift-off of resist or side wall re-deposition. The topographic spikes are rather small in height ($\lesssim$ 5 nm, i.e. $\lesssim$ 5% of total stack thickness) and cover $<$ 8% of the wire width. Notably, any surface defects are limited in their detriment to the magnetic contrast and skyrmion dynamics at the edge. Notably, we observe near-unitary skyrmion distribution across wire width with no preferential existence of skyrmions at the edge (see manuscript Fig. 3c).

S3. Skyrmion Nucleation & Properties

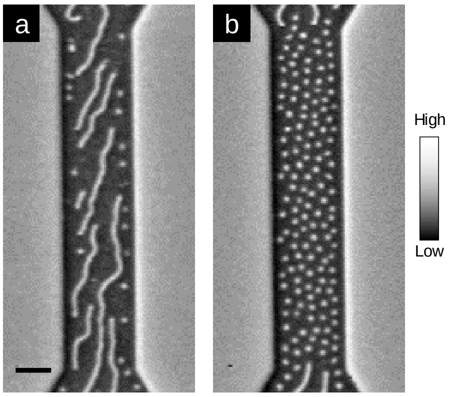

FIG. S3. **Skyrmion Creation Protocol.** MFM images (scale bar: 100 nm) of the wire at a representative OP field, after negative saturation, showing **(a)** initialized magnetic textures consisting of stripes and skyrmions, and **(b)** an array of skyrmions after following a pulse injection protocol to break up the stripes.

Skyrmion Creation. At lower magnetic fields, stripe domains may be stabilized along with skyrmions. To avoid ambiguity, we established a protocol involving current-induced fission of stripes[7] to ensure that the wire consists of solely skyrmions for dynamics studies. The protocol involved injecting current pulses with gradually increasing magnitude J, and observing MFM images for changes to the magnetic configuration. This enabled the selection of a minimum J for skyrmion creation while mitigating physical device damage or skyrmion annihilation due to heating. The pulsing continued at this J until no skyrmions were created with further pulsing. This protocol works only above a certain magnetic field threshold wherein skyrmions are metastable[7] – below which no amount of pulsing could create a skyrmion configuration. Therefore field values below the threshold were not included in this study.

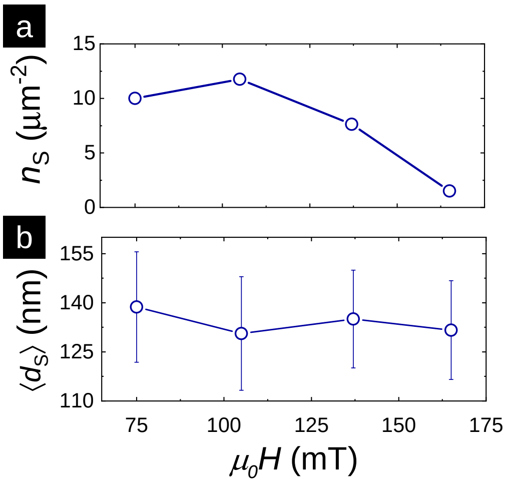

FIG. S4. **Skyrmions Properties.** **(a)** Density n_S, and **(b)** field-of-view average size $\langle d_S \rangle$ – of skyrmion configurations as a function of OP field after *ex situ* saturation at -400 mT followed by the skyrmion creation protocol (see above).

Skyrmion Properties. The skyrmion density (n_S, Fig. S4a) and field-of-view average size ($\langle d_S \rangle$, Fig. S4b) were measured from MFM images of skyrmion lattice configurations stabilized at various OP applied fields of 75 to 165 mT (detailed in manuscript Fig. 1). The n_S peaks at $\sim 13\,\mu\text{m}^{-2}$ under an OP applied field of 105 mT, followed by a gradual decline to $\sim 2\,\mu\text{m}^{-2}$ with increasing field. Meanwhile, the $\langle d_S \rangle$ shows a monotonic $\sim 6\%$ decrease from 140 to 132 nm over the range of applied fields. The large variance of skyrmion sizes at each

field ($\sim 80 - 200$ nm, see manuscript Fig. 4) enables statistically significant analysis of the size dependence of skyrmion dynamics.

S4. Skyrmion Motion Tracking Protocol

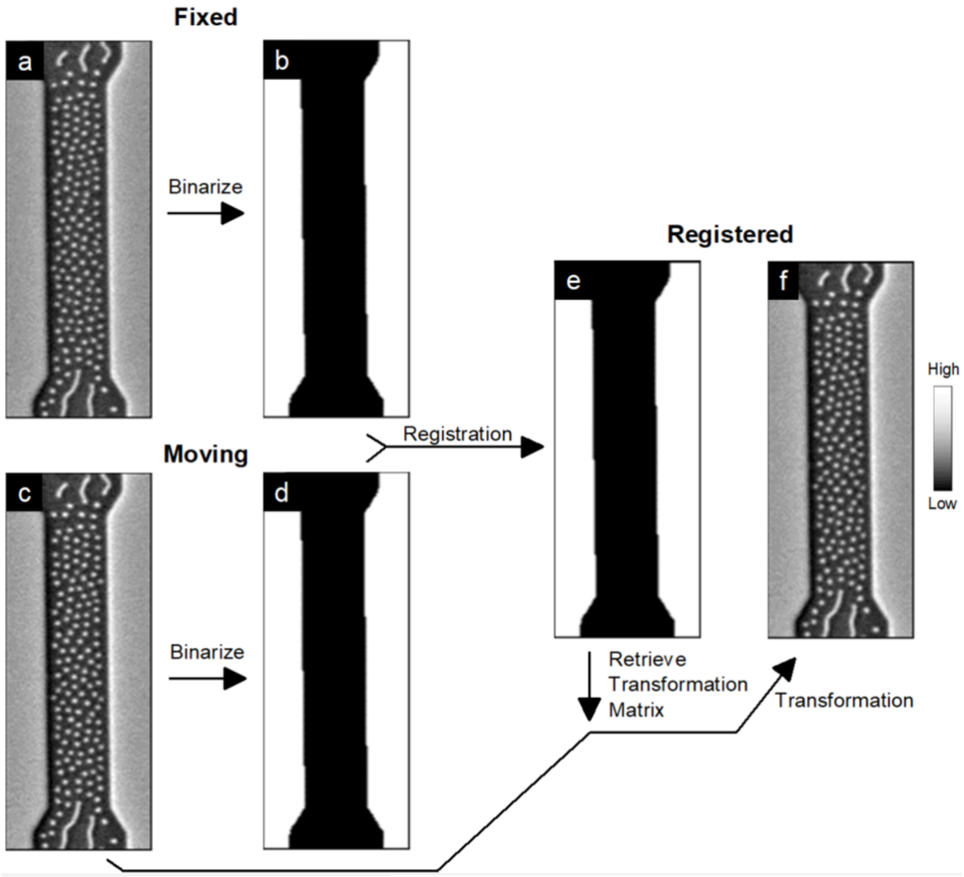

FIG. S5. **Registration of MFM Images. (a-b)**Reference MFM image (a) and the corresponding reference binary image (b) of the wire device. **(c-d)** Target MFM image (c) and the corresponding target binary image (d). **(e)** The resulting registered binary image derived from the registration of target (d) to reference (b). **(f)** The transformed MFM image of (c) based on the transformation matrix retrieved from (e).

Image Correction & Registration. The MFM images were initially processed using common scanning probe microscopic (SPM) image correction techniques including plane surface subtraction and row alignment. For the tracking of skyrmion motion, the wire device needs to be imaged at an identical position before and after each applied pulse. Since drifts inherent to SPM cannot be fully eradicated in experiments, image registration – i.e. post alignment of the image to a reference – is required to correct for slight position shifts. One means of registration is to align the device topography in each dataset. However, topographic artifacts due to fluctuations in feedback and scan speed deem such a technique unreliable in practice. Instead we adopt a less rigorous approach by using the MFM channel – which is less susceptible to scan speed issues and feedback fluctuations. To remove any potential registration bias from the domain patterns, the MFM images from the reference (fixed) and the target (moving) dataset are binarized and any hole present is filled up (Fig. S5a-d). Following this, the transformation matrix (TF) aligning the moving to the fixed binary data (Fig. S5e) is retrieved and applied to the moving MFM data (Fig. S5c) to obtain a registered MFM data (Fig. S5f).

Cumulative Registration. The intensity-based image registration was implemented using the Image Processing Toolbox in MATLAB® assuming a 2D affine transformation. Since the drifts from consecutive scans are relatively minor - evident in Fig. S5 a, negligibly small downward translation between the moving and fixed image, registrations are carried out on consecutive images instead of the reference dataset. The effective TF (ETF) aligning each dataset to the reference is then obtained by multiplying TFs aligning prior images

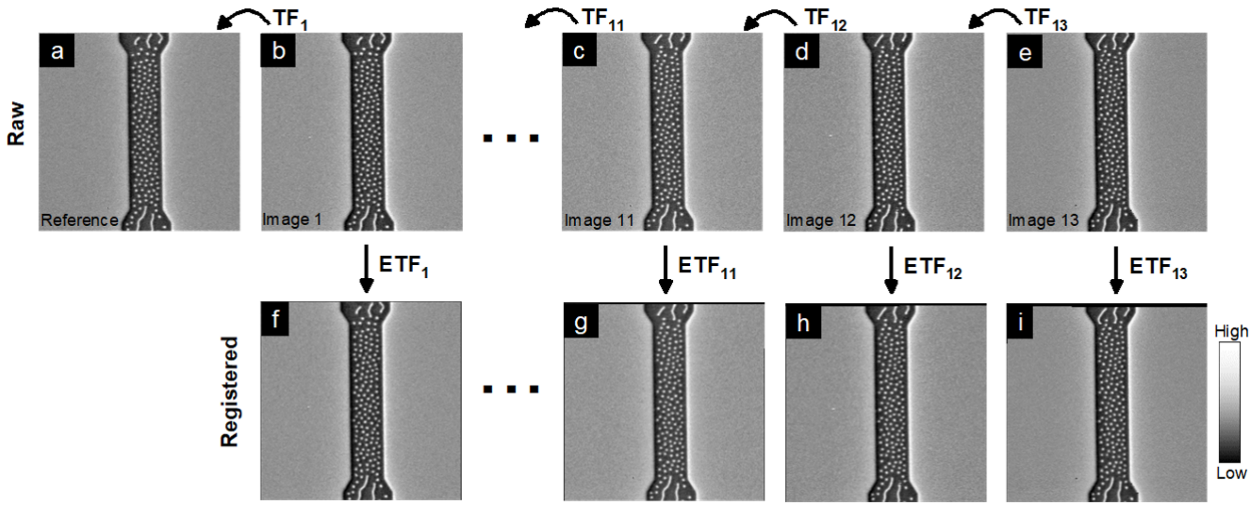

FIG. S6. **Cumulative Registration of Consecutively Acquired MFM Images.** **(a-e)** Set of consecutively acquired MFM images where TF_x is the transformation matrix aligning image x to image $(x-1)$. **(f-i)** Registered images of (b-e) aligned to reference image (a) where ETF_x is the effective transformation matrix defined in Eq. S3.

cumulatively, defined as

$$ETF_x = \prod_{1}^{x} TF_i \tag{S3}$$

As shown in Fig. S6, Image 1 aligns to the Reference via TF_1; Image 2 aligns to the Reference via TF_2 (TF between 1st and 2nd image) $\times TF_1$; Image 3 aligns to the Reference via $TF_3 \times TF_2 \times TF_1$ and so on. This registration protocol rigorously eliminates any erroneous registration results especially when a long time has elapsed between the acquisition of the reference and target MFM image.

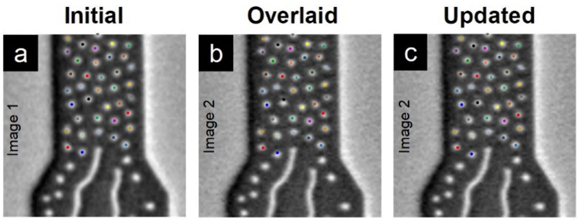

FIG. S7. **Skyrmion Tracking.** Initial skyrmion positions (shown as dots) overlaid on MFM images acquired **(a)** before pulse and **(b)** after pulse. **(c)** Final skyrmion positions overlaid on MFM image acquired after pulse. Each skyrmion is uniquely marked with a colored marker.

Skyrmion Tagging and Tracking. Upon generating skyrmions in the wire device (skyrmion nucleation protocol described in § S3), the skyrmions in the MFM image are identified and uniquely tagged. The identification of skyrmions is noticeably easier here as it does not involve differentiating stripes and skyrmions. To aid tracking, the previous skyrmion tags are overlaid on the next MFM image obtained after a pulse is injected. The skyrmion tags are updated from their previous positions by systematically assigning each tag to their respective new skyrmion position. It is crucial that the assignment of new positions follows a systematic top-down (or bottom-up) direction, ensuring each tag is assigned to the nearest skyrmion as much as possible. The tracking process is repeated for every pulse. The tracking protocol is rigorous only if the number of skyrmions remain nearly unchanged during pulsing. Importantly, any large fluctuation in numbers (skyrmion annihilation/creation) indicates a high likelihood of device damage due to substantial current-induced heating. Consequently, experiments were terminated or discarded from analysis in cases where skyrmion numbers showed drastic changes.

S5. Quantifying Skyrmion Dynamics Parameters

Retrieval of Skyrmion Size. To reduce bias arising from the choice of fit window size, we adopt a two-step fit process to obtain the d_S. The first fit iteration allows the estimation of the fitting parameters which are used to optimize the fitting window length to 1.5× of the estimated d_S. The second iteration uses the optimized window for fitting, with the estimated values as the initial guess. In both steps, the method of least squares was used to fit a Gaussian function, $f(x_i, y_j, \beta)$ to the observed signal, $z(x_i, y_j)$. To place the fit emphasis on the vicinity of the skyrmion, with radially decaying importance towards the corners of the fitting window, the residual, r_{ij} is therefore weighted by another Gaussian function, $f_\mathrm{weight}(x_i, y_j, \beta)$, defined as

$$r_{ij} = [f(x_i, y_j, \beta) - z(x_i, y_j)] \cdot f_\mathrm{weight}(x_i, y_j, \beta) \tag{S4}$$

$$f(x_i, y_j, \beta) = a \cdot \exp\left\{-\frac{(x_i - x_0)^2 + (y_j - y_0)^2}{2\sigma^2}\right\} + b \tag{S5}$$

$$f_\mathrm{weight}(x_i, y_j, \beta) = \exp\left\{-\frac{(x_i - x_0)^2 + (y_j - y_0)^2}{2\sigma^2}\right\} \tag{S6}$$

for set of fit parameters, $\beta = (a, b, x_0, y_0, \sigma)$. The d_S is hence defined by the linewidth of the fitted Gaussian, $f(\beta)$, given as $2\sqrt{(2\ln 2)}\sigma$.

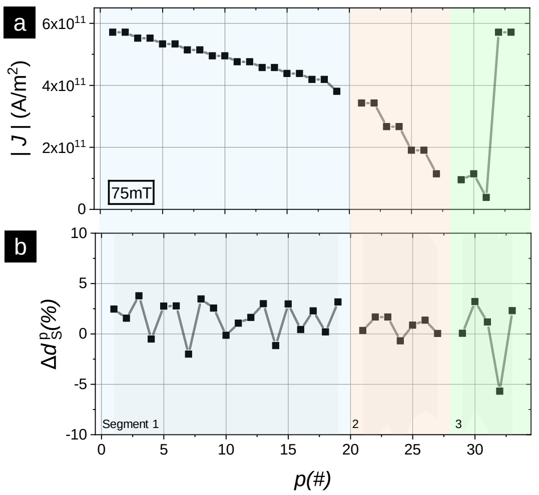

FIG. S8. **Change in Skyrmion Size. (a)** Magnitude of current density, $|J|$ and **(b)** corresponding change in skyrmion size, Δd^p_S for each applied pulse, p at OP field of 75mT.

Current-Induced Size Changes. Due to the linewise scanning nature of the MFM technique, images of the magnetic configuration can be acquired only before or after a current pulse is applied. The accuracy of the size analysis will therefore depend on the perturbation of d_S in response to the applied current pulse. We quantify this by recording the skyrmion size change, $\Delta d^p_{\mathrm{S}_i}$ after each pulse p, and quantifying the average change, Δd^p_S given as:

$$\Delta d^p_{\mathrm{S}_i}(\%) = \frac{d^p_{\mathrm{S}_i} - d^{p-1}_{\mathrm{S}_i}}{d^{p-1}_{\mathrm{S}_i}}, \ \mathrm{S}_i \text{ is the } i^\mathrm{th} \text{ skyrmion, and} \tag{S7}$$

$$\Delta d^p_\mathrm{S}(\%) = \frac{\sum_i^N \Delta d^p_{\mathrm{S}_i}}{N}, \ N \text{ is the total number of skyrmions.} \tag{S8}$$

We find that after each pulse p, $\mid \Delta d^p_\mathrm{S} \mid$ is $\lesssim 5\%$ for $|J|$ up to 6.0×10^{11} A/m^2 (Fig. S8). This indicates that the size analysis in manuscript Fig. 4 is valid to ±10 nm for larger d_S (< 200 nm) and to ±5 nm for smaller d_S (< 100 nm).

Dynamics Analysis. The parameters describing skyrmion motion – skyrmion velocity, v_S and deflection angle θ_S – are characterized with reference to the direction of J (see manuscript Fig. 2a). The v_S is given by the ratio of displacement of the skyrmion after the pulse injection to the effective pulse duration of 20 ns. The $\langle v_S \rangle$ detailed in manuscript Fig. 2 only includes skyrmions that are in motion, wherein any positional change of < 1 pixel (~ 50 nm) is considered as static. Meanwhile, the θ_S is wrapped in the range of $-90°$ to $90°$, with $0°$ being the direction of J. Specifically, in instances of θ_S less than $-90°$ or more than $90°$, a value of $180°$ is added or subtracted, respectively. This analysis was employed throughout the study with the exception of the plastic flow regime analyses (detailed in manuscript Fig. 3 and 4) where this angular projection is not valid – only skyrmions that move with J are considered. The data for v_S and θ_S analyses are binned and only bins with more than 5 skyrmions ($N_{\mathrm{cutoff}} > 5$) are considered for averaging. The N_{cutoff} is reduced to 1 for the skyrmion size analysis in manuscript Fig. 4 as the dataset is further analyzed for J dependence. The robustness of the analysis is addressed in § S7.

S6. Additional Data for Skyrmion Flow Dynamics

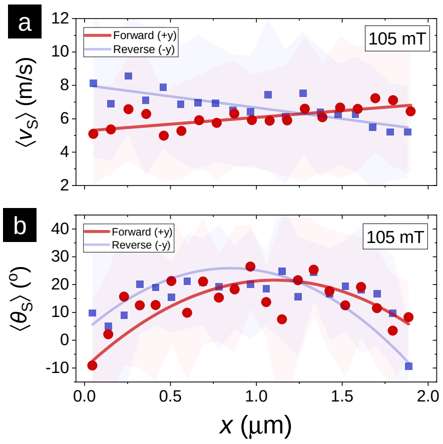

FIG. S9. **Confinement Effects on Skyrmion Flow Dynamics**. At $\mu_0 H \simeq 105$ mT, **(a)** the average velocity $\langle v_S(x) \rangle$ and **(b)** angular deflection $\langle \theta_S(x) \rangle$ for skyrmions in each x-bin for forward ($J \parallel +\hat{y}$, blue) and reverse ($J \parallel -\hat{y}$, red) motion. Solid lines are guides-to-the-eye, while shaded regions represent the standard deviation.

Edge Dependence of Velocity and SkHE. Analogous to manuscript Fig. 3d-e (75 mT), Fig. S9 shows the variation of $\langle v_S(x) \rangle$ and $\langle \theta_S(x) \rangle$ with transverse position x at 105 mT along the forward ($J \parallel +\hat{y}$) and reverse ($J \parallel -\hat{y}$) directions. Consistent with 75 mT observations, there is a linear decrease in $\langle v_S(x) \rangle$ by $\sim 30\%$ at the left edge of the wire in the forward motion (Fig. S9). Additionally, for both OP fields of 75 mT (manuscript Fig. 3e) and 105 mT (Fig. S9b), the $\langle \theta_S(x) \rangle$ increases from negative at $x = 0\,\mu$m to its maximally positive value at $x \sim 1\,\mu$m, and then decreases as x approaches $2\,\mu$m. The same variation for $\langle v_S(x) \rangle$ and $\langle \theta_S(x) \rangle$ are observed for both directions.

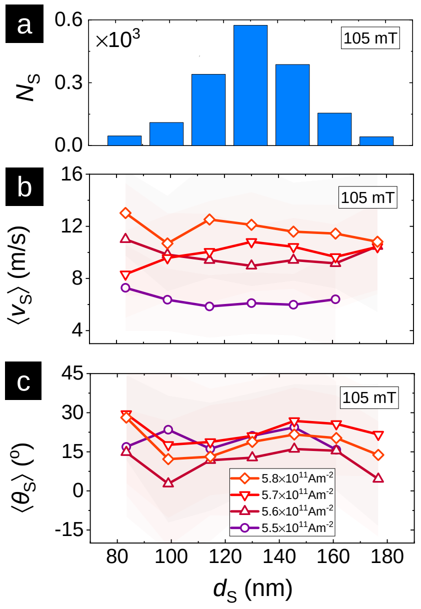

FIG. S10. **Skyrmion Size Effect on Skyrmion Flow Dynamics.** At $\mu_0 H \simeq 105$ mT, **(a)** binned histogram distribution of skyrmions in the plastic flow regime, N_S, based on their size, d_S – which varies over 80-180 nm. **(b-c)** Average velocity $\langle v_S \rangle$ (b) and angular deflection $\langle \theta_S \rangle$ (c) for skyrmions in each d_S-bin across the plastic flow regime ($J = (5.5 - 5.8) \times 10^{11}$ A/m^2). Shaded regions represent the standard deviation.

Size Dependence of Velocity and SkHE. We examine the influence of d_S, which varies over 80–180 nm (Fig. S10a), on the $\langle v_S \rangle$ (Fig. S10b) and $\langle \theta_S \rangle$ (Fig. S10c) for $J = (5.5 - 5.8) \times 10^{11}$ A/m^2 at 105 mT. Similar to

$\mu_0 H \simeq 75$ mT shown in manuscript Fig. 4d, $\langle v_S \rangle$ is insensitive to d_S. Meanwhile, the trend of $\langle \theta_S \rangle$ with d_S is less clear across the range of J, largely due to inconsistencies in the outermost bins. As these outermost bins have low skyrmion counts (5% of central bin), the size dependence analysis is improved in § S7 by imposing a minimum count criterion for each bin, resulting in a clearer d_S trend.

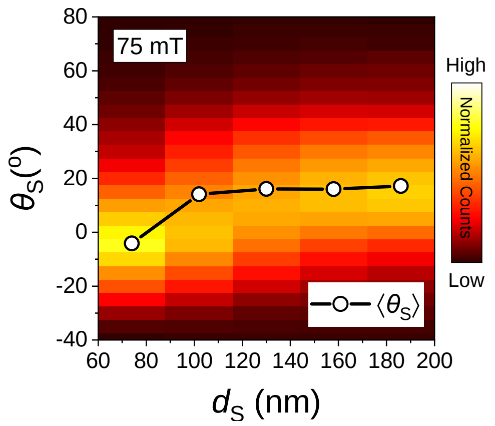

FIG. S11. **Skyrmion Size Effect on Skyrmion Flow Dynamics for Second Device.** 2D histogram color plot of θ_S against d_S for $\mu_0 H \simeq 75$ mT across all currents. The data were binned by d_S. Solid markers show the average deflection, $\langle \theta_S \rangle$, for each d_S bin.

Size Dependence of SkHE for Second Device. Here, we examine the size dependence of skyrmion dynamics in the plastic flow regime for a second device at $\mu_0 H \simeq 75$ mT and J over $5.5 - 5.7 \times 10^{11}$ A/m^2 (Fig. S11). The skyrmions are binned by their d_S which spreads over $70 - 190$ nm. As shown in the 2D histogram plot, we observe a discernible increase in $\langle \theta_S \rangle$ as d_S varies over 60–200 nm. The variation of $\langle \theta_S \rangle$ with d_S for this device is similar in magnitude to the results presented in the manuscript Fig. 4c.

S7. Binning Effects

We extend the analyses carried out in manuscript Fig. 3 and 4 – on the positional and skyrmion size dependence of skyrmion dynamics – with different bin parameters to verify the robustness of the trends presented.

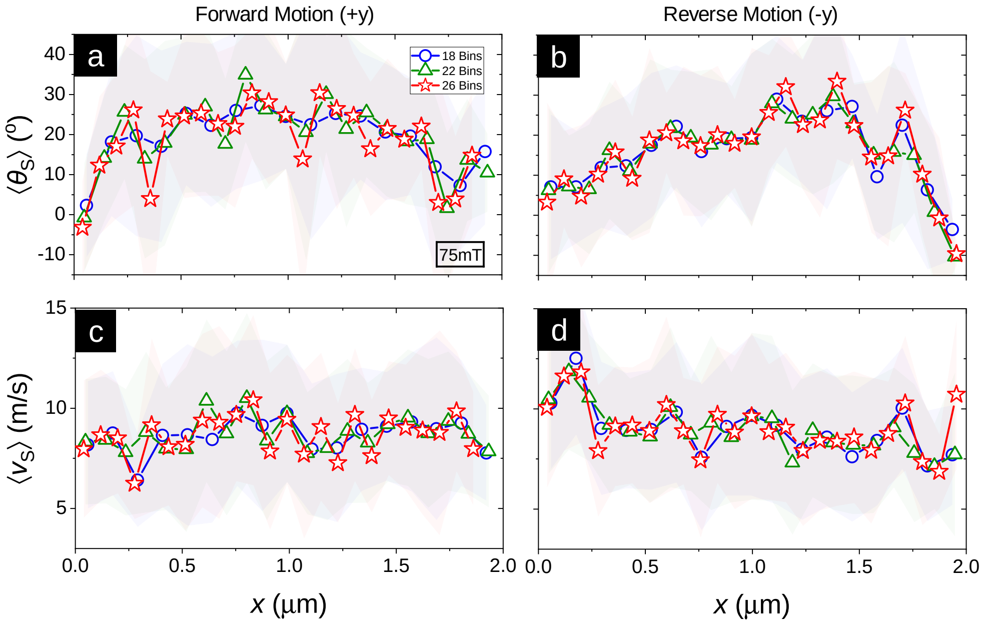

FIG. S12. **Bin Size Variation for Edge Effect Analysis. (a – d)** Positional dependence of $\langle\theta_{\mathrm{S}}\rangle$ for the (a) forward ($J \parallel +\hat{y}$) and and (b) reverse ($J \parallel -\hat{y}$) directions, and $\langle v_{\mathrm{S}}\rangle$ for the (c) forward ($J \parallel +\hat{y}$) and (d)reverse ($J \parallel -\hat{y}$) directions in the plastic flow regime with different bin sizes. Shaded regions represent the standard deviation.

Edge Effect Analysis. Fig. S12 shows the positional analysis (detailed in Fig. 3d, e) with bin sizes varied over a range of $\sim 40\%$ in both J directions. Both the $\langle\theta_{\mathrm{S}}\rangle$ (Fig. S12a, b) and $\langle v_{\mathrm{S}}\rangle$ (Fig. S12c, d) dependence of position clearly display the same trend for differing bin sizes. Notably, the current direction-dependent asymmetry of $\langle\theta_{\mathrm{S}}\rangle$ (Fig. S12a, b) and $\langle v_{\mathrm{S}}\rangle$ (Fig. S12c,d) with respect to position are also evident, as discussed in manuscript §D.

Skyrmion Size Effect Analysis. The same procedure of varying the bin sizes by $\sim 40\%$ was employed in the analysis of skyrmion size effect detailed in manuscript Fig. 4d, e. The observations of the average $\langle v_{\mathrm{S}}\rangle$ steadily increasing with J while relatively unchanged across d_{S} (Fig. 4d) are also evident here with different bin sizes (Fig. S13a – c). Similarly, the $\langle\theta_{\mathrm{S}}\rangle - d_{\mathrm{S}}$ relationship (Fig. S13d – f) also seems insensitive to variation in bin sizes.

Skyrmion Size Effect in the Plastic Flow Regime Analysis. The d_{S} dependence of $\langle v_{\mathrm{S}}\rangle$ (Fig. S14b) and $\langle\theta_{\mathrm{S}}\rangle$ (Fig. S14c) are largely reproduced within 4 standard deviations (4 Std) of the d_{S} distribution (Fig. S14a). Since the plastic flow dataset was further divided into different J for the analysis in manuscript Fig. 4c,d (also Fig. S13a, d), it is therefore reasonable that a smaller bin number would capture more accurately the size dependence trend at the tail of the size distribution (< 100 nm, Fig. S14a). To reinforce the skyrmion size dependence trends, we consolidated all the skyrmions moving in the plastic flow regime (i.e. removing the J categorization) and repeated the analysis. Using different bin sizes, Fig. S14c clearly shows a monotonic increase of $\langle\theta_{\mathrm{S}}\rangle$ with d_{S} within 4 Std of the d_{S} distribution (Fig. S14a) while $\langle v_{\mathrm{S}}\rangle$ as expected is mostly constant with d_{S} (Fig. S14b).

To improve the clarity of the skyrmion size analysis at 105 mT (Fig. S10b-c) , we impose a minimum count of 50 for each bin. This addresses potential inconsistencies at the outermost bins that may arise from individual outliers due to low counts. For both 75 and 105 mT, the additional analysis criterion results in clearer $\langle v_{\mathrm{S}}\rangle$,

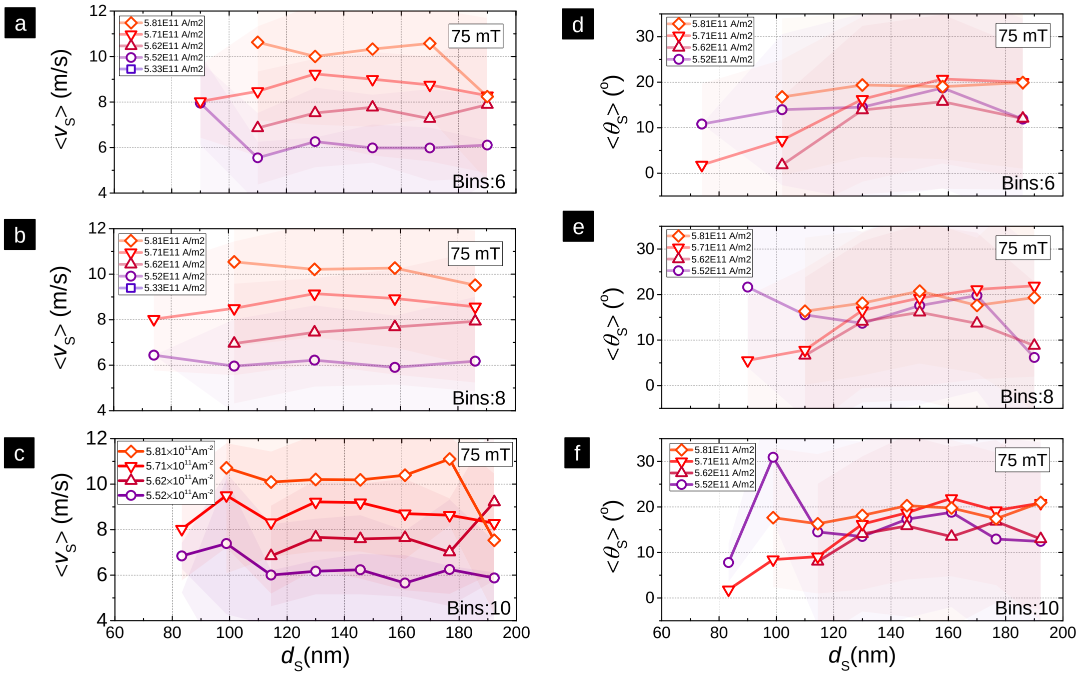

FIG. S13. **Bin Size Variation for Skyrmion Size Effect Analysis. (a–c)** $\langle v_S \rangle$ and **(d − f)** $\langle \theta_S \rangle$ in the plastic flow regime as a function of d_S for varying magnitudes of J with bin sizes of (a, d) 6, (b, e) 8, and (c, f) 10 bins. Shaded regions represent the standard deviation.

$\langle \theta_S \rangle$ vs d_S trends. Consistent with manuscript Fig. 4, $\langle v_S \rangle$ is insensitive (Fig. S15a, c) to d_S and $\langle \theta_S \rangle$ weakly increases with d_S (Fig. S15b, d).

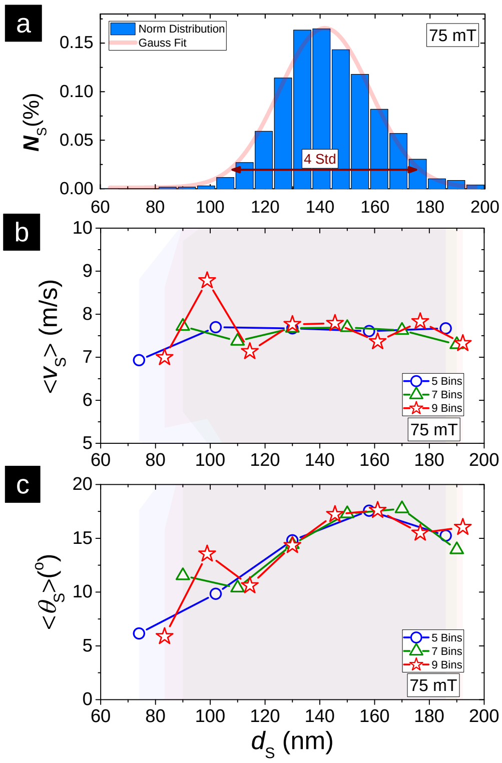

FIG. S14. **Bin Size Variation for Size Effect Analysis Consolidated for the Plastic Flow Regime.** **(a)** Normalized distribution of d_S in the plastic flow regime fitted with a Gaussian distribution. **(b − c)** Skyrmion size dependence of (b) $\langle v_S \rangle$ and (c) $\langle \theta_S \rangle$ averaged across all J in the plastic flow regime with different bin sizes. Shaded regions represent the standard deviation.

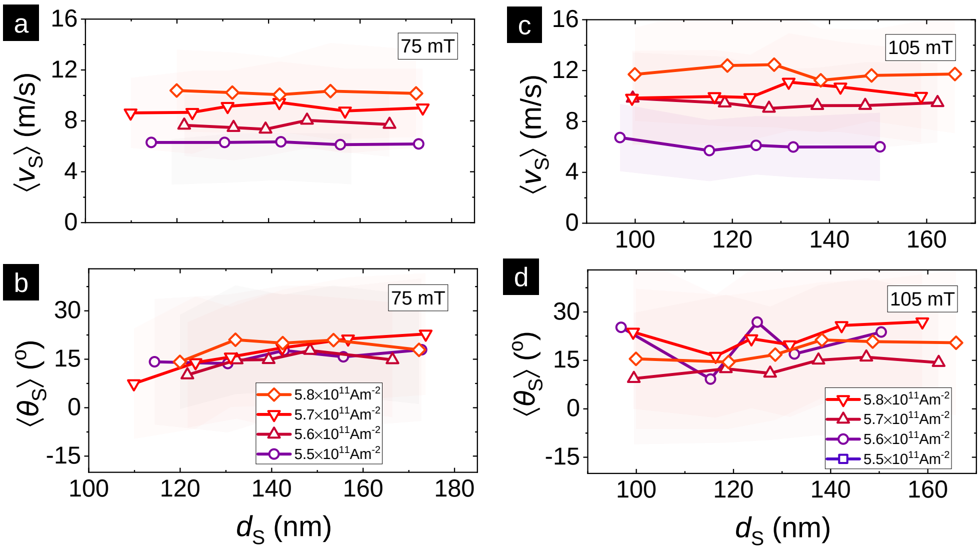

FIG. S15. **Skyrmion Size Effect Analysis with Minimum Counts Criterion.** **(a-d)** Average velocity $\langle v_S \rangle$ and angular deflection $\langle \theta_S \rangle$ with a minimum of 50 skyrmion counts in each d_S-bin across the plastic flow regime ($J = 5.5\text{–}5.8 \times 10^{11}\,\mathrm{A/m^2}$) at $\mu_0 H \simeq 75$ mT (a-b) and $\mu_0 H \simeq 105$ mT (c-d). Shaded regions represent the standard deviation.

S8. Micromagnetic Simulations

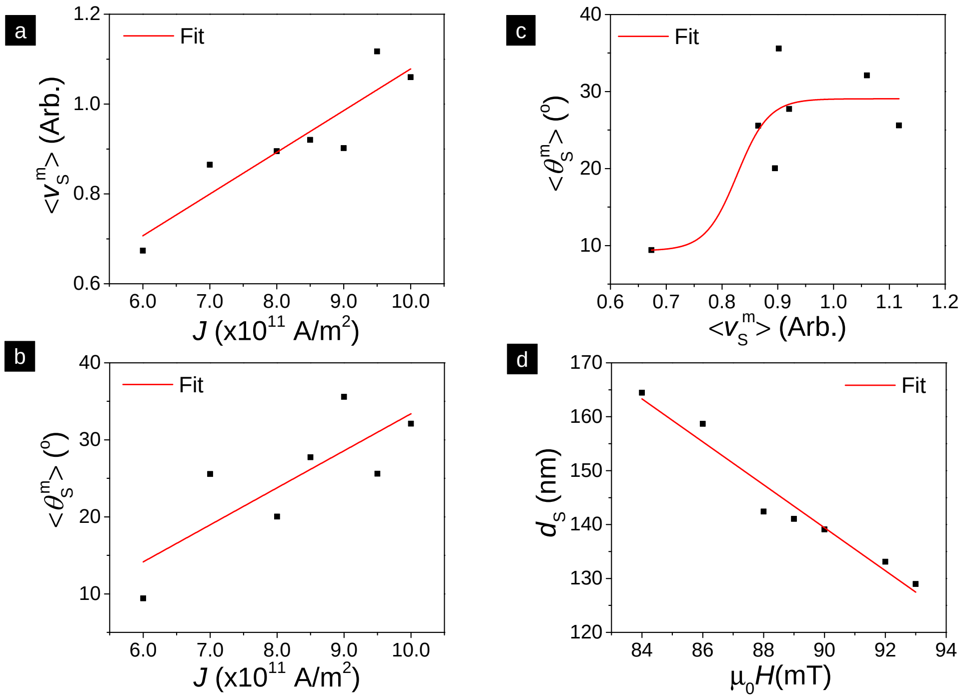

FIG. S16. **Micromagnetics Simulations.** **(a)** Average skyrmion velocity $\langle v_S^m \rangle$ and **(b)** angular deflection $\langle \theta_S^m \rangle$ for J varied from $(6-10)\times 10^{11}$ A/m^2 at a fixed OP field of 87 mT. **(c)** $\langle \theta_S^m \rangle$ as a function of $\langle v_S^m \rangle$ showing SkHE saturation in the flow regime at $J \gtrsim 9.0 \times 10^{11}$ A/m^2. **(d)** The variation of d_S as a function of OP field $\mu_0 H$, which was used to obtain the size dependence results.

Simulated Dynamics. Our defect free micromagnetic simulations show a monotonic linear increase in the simulated skyrmion velocity, $\langle v_S^m \rangle$ and angular deflection, $\langle \theta_S^m \rangle$ (Fig. S16a, b) with increasing J. In comparison, our experiments note an exponential $\langle v_S \rangle - J$ relationship, which additionally has a transition from the creep to flow regime (manuscript Fig. 2b), with differences arising likely due to pinning effects. Fig. S16c shows a saturation of the $\langle \theta_S^m \rangle$ at $\sim 28°$ at high $\langle v_S^m \rangle$, corresponding to J larger than 9.0×10^{11} A/m^2, comparable to the experimental skyrmion saturation, $\theta_S^{sat} \sim 22°$ in the flow regime. In ensuring consistency with experimental skyrmion dynamics analysis in the flow regime (manuscript Figs. 3 and 4), subsequent simulations on the d_S-dependence of average $\langle v_S^m \rangle$ and $\langle \theta_S^m \rangle$ (manuscript Fig. 5) were carried out at a sufficiently high J of 9.5×10^{11} A/m^2. Fig. S16d shows a linear reduction in simulated d_S by $\sim 30\%$ with increasing OP applied field. The specific range of OP fields was chosen to ensure that at most one or two sparse skyrmions were stabilized in the wire, which precludes any influence of skyrmion-skyrmion and skyrmion-stripe interactions on the simulated $\langle v_S^m \rangle$ and $\langle \theta_S^m \rangle$.

Comparison of Micromagnetic Simulations in Published Works. We summarize our grain-free micromagnetic simulations and relevant granular micromagnetic simulation works, which have incorporated pinning effects on skyrmion dynamics, for the ease of comparison with our experimental results (Tbl. S1). It is worth noting that neither our grain-free micromagnetic simulations nor the granular micromagnetic simulations can fully explain our key experimental observations - the skyrmion size dependence on velocity (v_S-d_S) and Hall angle (θ_S-d_S). This is likely because the pinning landscapes produced by the current granular implementation of disorder in micromagnetic simulations are not sufficiently representative of the true landscapes in disordered magnetic multilayers.

	v_S-J	θ_S-v_S	v_S-d_S	θ_S-d_S
**Experimental Results in Manuscript**	Exponential increase	S-curve	Constant	Weak increase
**Grain Free Micromagnetic Simulations in Manuscript**	Increase	S-curve	Increase to saturation	Decrease
**Granular Micromagnetic Simulations in Kim, J-V. et al.**[8]	Exponential increase	N.A.	N.A.	N.A.
**Granular Micromagnetic Simulations in Legrand, W. et al.**[9]	Exponential increase	N.A.	Increase to saturation	N.A.
**Granular Micromagnetic Simulations in Juge, R. et al.**[?]	Exponential increase	Increase to saturation	N.A.	N.A.

TABLE S1. **Published Micromagnetic Simulations.** Comparison of the Skyrmion Dynamics Trends Reported in Our Experiments and Grain-Free Micromagnetic Simulations with Published Micromagnetic Simulation Results

S9. Experimental Skyrmion Dynamics Characteristics in Published Works

In Tbl. S2, we present a tabulated comparison of the multilayer stacks used in our work, the associated skyrmion properties, and the observed dynamic characteristics, as compared with published results on multilayer skyrmions.

**Stack Composition**	**Skyrmion Sizes (d_S) & Densities (n_S)**	**Motion Regime**	**Max Velocity, v_S^{max} (m/s)**	**Max Deflection, θ_S^{max} (°)**	**Wire Edge (x) Dependence**		**Skyrmion Size (d_S) Dependence**	
					v_S	θ_S	v_S	θ_S
**Ferromagnetic**								
**[Pt/Co/MgO]$_{15}$**	d_S : 80-200 nm<br>n_S : 2-13 μm^{-2}	Stochastic<br>Creep<br>**Flow**	24	22°<br>(saturated)	~ 20%<br>reduction	Ambipolar<br>+22° → -5°	Independent<br>(flow)	Weak, linear<br>(flow)
[Pt/Co/Ir]$_{10}$ [9]	d_S ~ 100 nm<br>n_S ~ 3 μm^{-2}	Stochastic	1	N.A.	-	-	-	-
[Pt/CoFeB/MgO]$_{15}$ [10]	d_S ~ 113 nm<br>n_S ~ 1 μm^{-2}	Creep	105	32°	-	-	-	Inverse<br>(creep)
Pt/FM/Au/FM/Pt<br>FM:Ni/Co/Ni [11]	d_S ~ 150-200 nm<br>n_S ~ 2 μm^{-2}	Stochastic<br>Creep	65	N.A.	-	-	-	-
[Pt/CoFeB/MgO]$_{15}$ [12]	d_S ~ 200-300 nm<br>n_S ~ 3 μm^{-2}	Creep	110	N.A.	-	-	-	-
[Pt/Co/Ta]$_{15}$ [12]	d_S ~ 200-300 nm<br>n_S ~ 3 μm^{-2}	Creep	50	N.A.	-	-	-	-
Ta/CoFeB/TaO$_x$ [13]	d_S ~ 1000 nm<br>n_S < 1 μm^{-2}	Stochastic<br>Creep<br>**Flow**	0.75	35°	Reduction	-	-	-
Pt/Co/MgO [14]	d_S ~ 156 nm<br>n_S ~ 1.5 μm^{-2}	Flow	100	50°<br>(saturated)	-	-	-	-
[Pt/CoB/Ir]$_5$ [15]	d_S ~ 400 nm<br>n_S ~ 1.5 μm^{-2}	Creep<br>**Flow**	6	9°<br>(saturated)	-	-	Independent<br>(flow)	Independent<br>(flow)
**Ferrimagnetic**								
[Pt/GdFeCo/SiN] [16]	d_S ~ 10,000 nm<br>n_S < 1 μm^{-2}	Creep	N.A.	35°	-	-	-	-
[Pt/GdFeCo/MgO]$_2$ [17]	d_S ~ 180 nm<br>n_S ~ 3 μm^{-2}	Stochastic<br>Creep<br>Flow	50	25°<br>(saturated)	-	-	-	-

TABLE S2. **Experimental Results on Skyrmion Dynamics.** Comparison of experimental results on skyrmion dynamics presented in this manuscript with published results. Key comparisons include the properties of skyrmion configurations, motion regimes, skyrmion velocity, deflection angle and dependence of dynamics on distance from the wire edge (x) and skyrmion size (d_S).

* These authors contributed equally to this work
† These authors contributed equally to this work; hopin@imre.a-star.edu.sg
‡ anjan@imre.a-star.edu.sg

[1] Soumyanarayanan, A. *et al.* Tunable Room Temperature Magnetic Skyrmions in Ir/Fe/Co/Pt Multilayers. *Nature Materials* **16**, 898–904 (2017).
[2] Ho, P. *et al.* Geometrically Tailored Skyrmions at Zero Magnetic Field in Multilayered Nanostructures. *Physical Review Applied* **11**, 024064 (2019).
[3] He, S. *et al.* A Versatile Rotary-Stage High Frequency Probe Station for Studying Magnetic Films and Devices. *Review of Scientific Instruments* **87**, 074704 (2016).
[4] Lourembam, J., Ghosh, A. & Zeng, M. Thickness-Dependent Perpendicular Magnetic Anisotropy and Gilbert Damping in Hf/Co20Fe60B20/MgO Heterostructures. *Physical Review Applied* **10**, 044057 (2018).
[5] Kittel, C. On the Theory of Ferromagnetic Resonance Absorption. *Physical Review* **73**, 155 (1948).
[6] Celinski, Z. & Heinrich, B. Ferromagnetic resonance linewidth of Fe ultrathin films grown on a bcc Cu substrate. *Journal of Applied Physics* **70**, 5935 (1991).
[7] Tan, A. K. C. *et al.* Skyrmion generation from irreversible fission of stripes in chiral multilayer films. *Phys. Rev. Materials* **4**, 114419 (2020).
[8] Kim, J.-V. & Yoo, M.-W. Current-driven skyrmion dynamics in disordered films. *Applied Physics Letters* **110**, 132404 (2017).
[9] Legrand, W. *et al.* Room-Temperature Current-Induced Generation and Motion of sub-100 nm Skyrmions. *Nano Letters* **17**, 2703–2712 (2017).
[10] Litzius, K. *et al.* Skyrmion Hall effect revealed by direct time-resolved X-ray microscopy. *Nature Physics* **13**, 170–175 (2016).
[11] Hrabec, A. *et al.* Current-induced skyrmion generation and dynamics in symmetric bilayers. *Nature Communications* **8**, 15765 (2017).
[12] Woo, S. *et al.* Observation of room-temperature magnetic skyrmions and their current-driven dynamics in ultrathin metallic ferromagnets. *Nature Materials* **15**, 501–506 (2016).
[13] Jiang, W. *et al.* Direct observation of the skyrmion Hall effect. *Nature Physics* **13**, 162–169 (2016).
[14] Juge, R. *et al.* Current-Driven Skyrmion Dynamics and Drive-Dependent Skyrmion Hall Effect in an Ultrathin Film. *Physical Review Applied* **12**, 044007 (2019).
[15] Zeissler, K. *et al.* Diameter-independent skyrmion Hall angle observed in chiral magnetic multilayers. *Nature Communications* **11**, 428 (2020).
[16] Hirata, Y. *et al.* Vanishing skyrmion Hall effect at the angular momentum compensation temperature of a ferrimagnet. *Nature Nanotechnology* **14**, 232–236 (2019).
[17] Woo, S. *et al.* Current-driven dynamics and inhibition of the skyrmion Hall effect of ferrimagnetic skyrmions in GdFeCo films. *Nature Communications* **9**, 959 (2018).
